# Report of Case of Pellagra

**Published:** 1910-10

**Authors:** A. D. McLain

**Affiliations:** Salem, Ala.


### REPORT OF CASE OF PELLAGRA.*

By Dr. A. D. McLain, Salem, Ala.

Case No. 1, Mrs. C. H., age 75, who presented the following history:

Family history.—Father and mother lived to be quite old, cause of death unknown, three brothers and two sisters living and in good health. One brother and two sisters died quite young.

Past history.—Had always been in good health up until the time of present illness, which began in the spring of 1908. At that time she though she was suffering from indigestion, and in the summer was troubled a great deal with diarrhoea, but as cold weather came on she got better.

On June 14th, 1909, I was called to see her, and found her suffering with the following symptoms:

Symptoms.—Lassitude, great fatigue, vertigo, diarrhoea, general disturbance of digestion, and mental despondency, but with no signs of eruption of hands or feet or any part of the body. In about six or eight weeks the skin on hands and lower part of fore-arm assumed a reddish color, soon developed a variable degree of thickening, and to these changes were added burning and itching sensations, and later on became sore and remained that way.

Temperature normal most all the time, and urine normal along about the first; did not test later.

As soon as hands became involved I made a positive diagnosis and put her on the arsenic treatment, but if it did any good I couldn't tell it. Patient became constipated about the middle of September, and it was as hard to get the bowels to act as it had been to keep them from acting before. About the same time she became insane and did not know anything or any body. At times she refused to take any nourishment on account of muscles that are brought into action during the act of swallowing, being partially paralyzed. On November 7th, 1909, patient died.

Case No. 2, Mrs. H., age 47, who presented the following history:

Family history.—Father got killed at age of 49, mother lived to be very old; cause of death unknown. Two brothers, both living and in good health. One sister living, and two dead. Died very young.

Past history.—Patient had always been in good health until about three years before last illness.

On June 14th, the same day that I was called to see case No. 1, I was called to see case No. 2, although they lived eight miles apart and were not acquainted with each other. I found identically the same symptoms with the exception of pain in epigastrium in case No. 2, while case No. 1 had no pain at all during her afflictions. The mucous membrane of vagina and rectum were very much involved in case No. 2, and case No. 1 was not bothered with that at all.

Eruption appeared on hands in both cases the same way, only exfoliation was more marked in case No. 2 than in case No. 1. Patient was crazy at times, urine contained a trace of albumen, temperature normal most all the time.

Treatment.—Put her on the arsenic treatment, but without any result, and on August 20th, 1909, patient died.

I want to mention two other cases to which I was called in consultation with Doctors B. and H. Case No. 3, Mrs. M. D., age 60, who presented the same symptoms the same summer as did case No. 1, and died December, 1909.

